# A description of patient experience after undergoing an emergency laparotomy

**DOI:** 10.1186/s13017-026-00686-y

**Published:** 2026-04-22

**Authors:** Struan Ambrose, Louise Silva, Rachel John–Charles, Hwei Ng, Hannah Javanmard-Emamghissi, Sarah Abbas Mohammed, Rashid Murtaza, Alina Dietrich, Maggie Clark, Simon Baker, Victoria Redfern, Gillian Tierney, Susan Moug, Julie Cornish

**Affiliations:** 1 University of Glasgow, University Avenue, Glasgow, G12 8QQ Scotland; 2 Cardiff and Vale University Health Board, Heath Park, Cardiff, CF14 4XW Wales; 3 Department of Surgery, Royal Alexandra Hospital, Corsebar Road, Paisley, PA2 9PN Scotland; 4 College of Medical, Veterinary and Life Sciences, University of Glasgow, Glasgow, G12 8QQ Scotland; 5 University Hospitals of Derby and Burton NHS Foundation Trust, Derby, DE22 3NE England

**Keywords:** Emergency laparotomy, Postoperative outcomes, Survivorship

## Abstract

**Background:**

To date, emergency laparotomy outcomes have centred on mortality, while survivorship remains inadequately defined and underexplored. It is expected that survivors are likely to experience significant short- and long-term biopsychosocial challenges and research is key to improving our understanding and identifying areas for improvement. This study aims to describe the emergency laparotomy postoperative pathway and evaluate short- and long-term outcomes.

**Methods:**

This was a retrospective observational study across three NHS hospitals in Scotland, England and Wales. All patients had undergone emergency laparotomy between December 2017 and January 2019 according to the established National Emergency Laparotomy Audit criteria. Inpatient and post-discharge data were collected, including in-hospital complications, planned surgical follow-up, and unplanned follow-up (representation, readmission, and primary care referrals).

**Results:**

Over the 14-month period, 557 patients were included (Scotland *n* = 199, Wales *n* = 252, and England *n* = 106), with 51.7% female and a median age of 65 years (IQR, 52–75 years). A total of 64.5% of patients had planned surgical follow-up, with a median interval of 9 weeks (IQR, 5–15 weeks). Within 30 days of discharge, 19.3% of patients represented to hospital and 13.3% required readmission. Within two years of discharge, 23.2% had primary care referrals to specialists, primarily general surgery and gastroenterology.

**Conclusion:**

Emergency laparotomy is associated with a complex recovery trajectory, with significant variation described. Many patients did not receive planned follow-up, and among those who did, timelines vary from 1 to 4 months. Given the high rates of unplanned follow-up, defining and improving survivorship is urgently needed for this vulnerable patient population.

**Supplementary Information:**

The online version contains supplementary material available at 10.1186/s13017-026-00686-y.

## Introduction

Major non-elective abdominal surgery (emergency laparotomy, EmLap) is a commonly performed surgical procedure, with approximately 30,000 patients undergoing EmLap within the National Health Service (NHS) each year [1, 2]. EmLap is indicated for a variety of abdominal pathologies, which can be broadly divided into obstruction, perforation, infection, ischaemia, and bleeding. The majority of patients are older adults, a significant proportion are living with frailty, and most are American Association of Anesthesiologists (ASA) III or higher [3]. This results in the EmLap population often being classified as high risk, making good perioperative care essential to minimise poor patient outcomes.

EmLap carries a higher risk of 30-day mortality compared to elective surgery, but as a result of national audits and the accompanying standards of care that sites have implemented, this has significantly reduced [1, 2, 4, 5]. Despite this, a substantial knowledge gap remains with survivorship and follow-up pathways among EmLap populations, as highlighted in recent systematic reviews [6, 7]. This contrasts sharply with the elective surgical population, which has evidence-based accepted pathways and supportive networks, especially if there is a cancer diagnosis [8, 9].

Survivorship has been reported as a priority in patients’ decision to undergo EmLap, with survivors likely to face a range of immediate and long-term biopsychosocial challenges [10]. These priorities differed from perioperative specialists, with readmission to hospital, recovery expectations, and follow-up plans highlighted. This study builds on these patient findings by aiming to report on the postoperative pathway following EmLap through exploring a range of inpatient and post-discharge indicators across three NHS centres.

## Methods

### Study design and hospitals

This was a retrospective observational study conducted across three NHS hospitals and reported using the Strengthening the Reporting of Observational Studies in Epidemiology (STROBE) statement [11] (Appendix 1). The study cohort comprised adult patients who underwent EmLap between December 2017 and January 2019 (14 months duration), as defined by the inclusion and exclusion criteria of National Emergency Laparotomy Audit (NELA) [1]. To optimise geographical diversity, the participating hospitals were Royal Alexandra Hospital (Site 1, Paisley, Scotland), Royal Derby Hospital (Site 2, Derby, England), and University Hospital Wales (Site 3, Cardiff, Wales). All participating sites are NHS teaching hospitals, each performing more than 200 EmLaps annually and operating a rotational on-call system delivered by upper and lower gastrointestinal consultant surgeons. No site reported a departmental follow-up protocol, with any planned follow-up decided by the responsible consultant at the time of discharge. The study protocol was reviewed by each site’s local Research and Development departments, which determined that formal ethical approval was not required.

### Patient identification and data collection

Patients were identified using local NELA and Emergency Laparotomy and Laparoscopic Scottish Audit (ELLSA) databases at each site [1, 2]. Baseline characteristics were extracted from these databases and included: age, sex, cancer diagnosis, stoma formation, length of hospital stay (prolonged stay defined as > 14 days), in-hospital mortality. Patients’ electronic health record was accessed to report on: route of admission to hospital, history of mental health conditions, history of chronic pain, in-hospital complications after EmLap. Route of admission was categorised according to the pathway by which EmLap was initiated: referral from general practice, presentation via emergency department, or inpatient referral (defined as patients already hospitalised for another indication who subsequently deteriorated requiring EmLap during the same admission). To characterise the postoperative pathway, the following post-discharge indicators were recorded: discharge destination, planned surgical outpatient follow-up, unplanned follow-up events. Discharge destination was classified as discharge home or discharge to another service (rehabilitation facility, mental health hospital, care home, nursing facility, or transfer to another hospital). Planned surgical outpatient follow-up was defined as a follow-up appointment arranged at discharge, with the time it was scheduled for recorded (up to two years after discharge). Unplanned follow-up events included: representation to the hospital and readmission to the hospital (both within 30 days of discharge), and referrals from primary care to specialist services (within 2 years of discharge). All unplanned follow-up events were only included when directly attributable to the EmLap.

### Statistical analysis

Analyses were conducted on available data, with observations containing missing values excluded from the relevant analyses. Continuous variables (age and length of stay) demonstrated non-normal distributions on visual inspection and are presented as medians with interquartile ranges (IQR). Multivariable logistic regression was used to examine associations between patient characteristics and unplanned follow-up events (defined as at least one unplanned event). The model included age (per 10-year increase), sex, mental health history, chronic pain history, cancer diagnosis, stoma formation, and prolonged hospital stay. Results are reported as adjusted odds ratios (aOR) with 95% confidence intervals. Statistical analyses were performed using R Statistical Software (v4.4.1; R Core Team 2021).

## Results

### Patient characteristics

A total of 594 patients met the inclusion and exclusion criteria, of which 37 were subsequently excluded because of missing data across significant variables (age, sex, data of hospital admission). The remaining 557 patients were included in the study, with distribution across the three sites (Site 1 = 199, 36%; Site 2 = 106, 19%; Site 3 = 252, 45%) (Table 1). The median age was 65 years (IQR, 52–75 years) with 51.7% female. A history of mental health conditions was reported in 26.0% of patients, and chronic pain in 7.2%. The median length of hospital stay was 12 days (IQR, 8–21 days), and in-hospital mortality was 8.1%, both of which are comparable to national EmLap data [1, 2].

**Table 1:** Baseline characteristics and perioperative outcomes

	Site 1 (*N* = 199)	Site 2 (*N* = 106)	Site 3 (*N* = 252)	Total (*N* = 557)
Age – yr, median (IQR)	66 (54–75)	61.5 (49–73)	65.5 (51–76)	65 (52–75)
Sex				
Male – no. (%)	94 (47.2)	48 (45.3)	127 (50.4)	269 (48.3)
Female – no. (%)	105 (52.8)	58 (54.7)	125 (49.6)	288 (51.7)
Route of Admission				
General practice – no. (%)	90 (45.2)	26 (24.5)	153 (60.7)	269 (48.3)
Emergency department – no. (%)	96 (48.2)	57 (53.8)	59 (23.4)	212 (38.1)
Inpatient at time of emergency laparotomy– no. (%)	12 (6.0)	21 (19.8)	20 (7.9)	53 (9.5)
Past Medical History				
Mental health – no. (%)	38 (19.1)	16 (15.1)	91 (36.1)	145 (26.0)
Chronic pain – no. (%)	12 (6.0)	14 (13.2)	14 (5.6)	40 (7.2)
Perioperative Outcomes				
Cancer diagnosis – no. (%)	33 (16.6)	17 (16.0)	40 (15.9)	90 (16.2)
Stoma formation – no. (%)	85 (42.7)	54 (50.9)	100 (39.7)	239 (42.9)
Length of hospital stay – d, median (IQR)	11 (7–21)	14 (8–20)	12 (8–22)	12 (8–21)
In-hospital mortality – no. (%)	17 (8.5)	3 (2.8)	25 (9.9)	45 (8.1)

The following data was missing: route of admission (Scotland [*n* = 1, 0.5%], England [*n* = 2, 1.9%], Wales [*n* = 20, 7.9], Total [*n* = 23, 4.1%]); mental health (Scotland [*n* = 1, 0.5%], Wales [*n* = 39, 15.5%], Total [*n* = 40, 7.2%]); chronic pain (Scotland [*n* = 1, 0.5%], Wales [*n* = 40, 15.9%], Total [*n* = 41, 7.4%]); cancer diagnosis (Scotland [*n* = 2, 1.0%], Wales [*n* = 5, 2.0%], Total [*n* = 7, 1.3%]); stoma formation (Scotland [*n* = 2, 1.0%], Wales [*n* = 17, 6.7%], Total [*n* = 19, 3.4%]); length of hospital stay (Scotland [*n* = 13, 6.5%], England [3, 2.8%], Total [*n* = 16, 2.9%]). IQR, interquartile range.

### EmLap patient pathway

#### Index hospitalisation

The most common pathways by which EmLap was initiated were referral from General Practice (48.3%) and presentation via the Emergency Department (38.1%). Stoma formation was required in 42.9% of patients and 16.2% of patients had a new cancer diagnosis. In-hospital complications occurred in 30.9% of patients, with 8.3% experiencing multiple; the most frequently reported were ileus (43 patients, 7.7%), wound infection (39 patients, 7.0%), and lower respiratory tract infection (36 patients, 6.5%) (Table 2).

**Table 2:** In-hospital complications after emergency laparotomy

	Site 1 (*N* = 199)	Site 2 (*N* = 106)	Site 3 (*N* = 252)	Total (*N* = 557)
In-Hospital Complications				
In-hospital complications (patients) – no. (%)	69 (34.7)	59 (55.7)	44 (17.5)	172 (30.9)
> 1 In-hospital complications (patients) – no. (%)	0 (0.0)	13 (12.3)	33 (13.1)	46 (8.3)
In-Hospital Complications By Type				
Infective				
Wound infection – no. (%)	12 (6.0)	19 (17.9)	8 (3.2)	39 (7.0)
Intra-abdominal collection – no. (%)	10 (5.0)	16 (15.1)	2 (1.0)	28 (5.0)
Lower respiratory tract infection – no. (%)	14 (7.0)	12 (11.3)	10 (4.0)	36 (6.5)
Urinary tract infection – no. (%)	0 (0.0)	0 (0.0)	9 (3.6)	9 (1.6)
Cardiopulmonary				
Pulmonary oedema – no. (%)	1 (0.5)	2 (1.9)	0 (0.0)	3 (0.5)
Shortness of breath – no. (%)	2 (1.0)	2 (1.9)	1 (0.4)	5 (0.9)
Deep vein thrombosis/pulmonary embolism – no. (%)	1 (0.5)	0 (0.0)	1 (0.4)	2 (0.4)
Cardiac event (arrythmia/myocardial infarction) – no. (%)	3 (1.5)	7 (6.6)	5 (2.0)	15 (2.7)
Gastrointestinal and Nutritional				
Ileus – no. (%)	14 (7.0)	13 (12.3)	16 (6.3)	43 (7.7)
High output stoma – no. (%)	0 (0.0)	0 (0.0)	9 (3.6)	9 (1.6)
TPN requirement for any indication – no. (%)	8 (4.0)	3 (2.8)	0 (0.0)	11 (2.0)
Enteral nutrition – no. (%)	0 (0.0)	0 (0.0)	25 (9.9)	25 (4.5)
Parenteral nutrition – no. (%)	0 (0.0)	0 (0.0)	4 (1.6)	4 (0.7)
Renal and Metabolic				
Fluid overload – no. (%)	0 (0.0)	0 (0.0)	8 (3.2)	8 (1.4)
Acute kidney injury – no. (%)	4 (2.0)	2 (1.9)	12 (4.8)	18 (3.2)
Other				
Delirium – no. (%)	0 (0.0)	0 (0.0)	19 (7.5)	19 (3.4)
Blood transfusion – no. (%)	0 (0.0)	0 (0.0)	2 (1.0)	2 (0.4)

The following data was missing: patients with in-hospital complications (Scotland [*n* = 2, 1.0%], Total [*n* = 2, 0.4%]); patients with multiple in-hospital complications (Scotland [*n* = 2], Total [*n* = 2, 0.4%]). TPN, total parenteral nutrition.

#### Planned follow-up

**Fig. 1:**
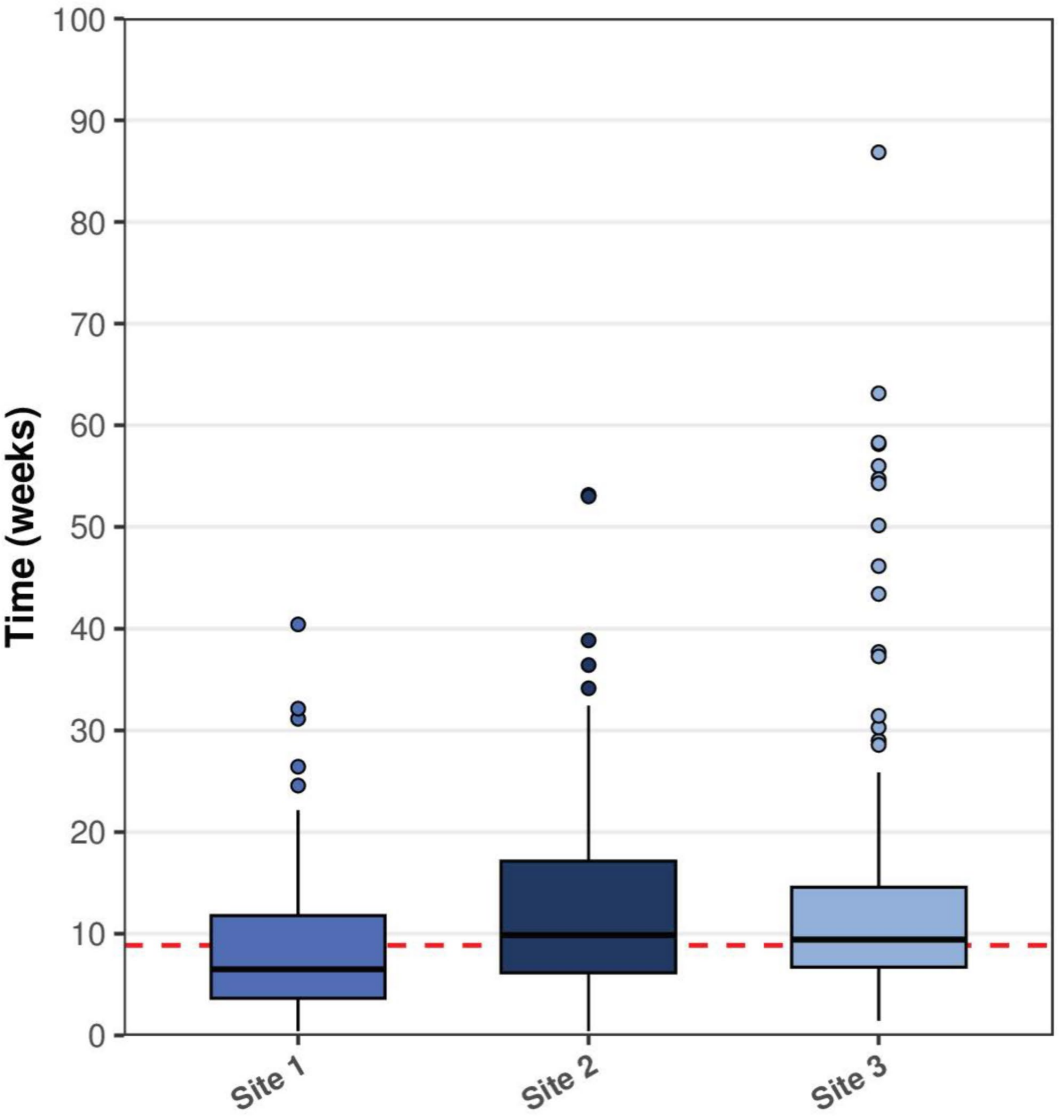
Time to surgical outpatient follow-up. Boxplots of the time (weeks) from hospital discharge to surgical outpatient follow-up at each participating site. The dashed red line represents the median time across all sites

Post-discharge outcomes were reviewed in the 512 patients who survived to hospital discharge (Table 3). The majority of patients were discharged home (466 patients, 91.0%). A total of 330 patients (64.5%) had planned surgical outpatient follow-up, with the median interval from discharge to follow-up being 9 weeks (IQR 5–15 weeks, range 0–87 weeks). The proportion was higher among patients with a new cancer diagnosis, of whom 82.9% had follow-up arranged at discharge. Planned surgical outpatient follow-up varied significantly by site (*p* = 0.04), with the highest rates at Site 2 (74.8%), compared with Site 3 (63.0%), and Site 1 (60.4%). The interval to follow-up also differed (Fig. 1), with a median of 6 weeks at Site 1 (IQR 4–12 weeks), 10 weeks at Site 2 (IQR 6–17 weeks), and 9 weeks at Site 3 (IQR 7–14 weeks). Further analysis at one site (Site 3) found further variation between individual consultants, with follow-up rates ranging from 54% to 88% and median times from 8 to 17 weeks. Equivalent consultant-level data was not available at the other two sites.

**Table 3:** Post-discharge emergency laparotomy indicators

	Site 1 (*N* = 182)	Site 2 (*N* = 103)	Site 3 (*N* = 227)	Total (*N* = 512)
Discharge Destination				
Discharge home – no. (%)	165 (90.7)	95 (92.2)	206 (90.7)	466 (91.0)
Discharge to rehabilitation facility – no. (%)	0 (0.0)	5 (4.9)	0 (0.0)	5 (1.0)
Discharge to mental health hospital – no. (%)	0 (0.0)	1 (1.0)	0 (0.0)	1 (0.2)
Discharge to care home – no. (%)	0 (0.0)	2 (1.9)	2 (0.9)	4 (0.8)
Discharge to nursing facility – no. (%)	0 (0.0)	0 (0.0)	4 (1.8)	4 (0.8)
Transfer to another hospital – no. (%)	10 (5.5)	0 (0.0)	4 (1.8)	14 (2.7)
Unplanned Follow-Up Events				
Representation to hospital – no. (%)	21 (11.5)	29 (28.2)	49 (21.6)	99 (19.3)
Readmission to hospital – no. (%)	20 (11.0)	20 (19.4)	28 (12.3)	68 (13.3)
Primary care referrals to specialists – no. (%)	16 (18.8)	18 (17.5)	85 (37.4)	119 (23.2)
Patients with ≥ 1 unplanned follow-up event – no. (%)	34 (18.7)	39 (37.7)	114 (50.2)	187 (36.5)
Planned Outpatient Follow-Up				
Surgical outpatient follow-up – no. (%)	110 (60.4)	77 (74.8)	143 (63.0)	330 (64.5)

The following data was missing: discharge destination (Scotland [*n* = 7, 3.8%], Wales [*n* = 11, 4.8%], Total [*n* = 18, 3.5%]).

#### Unplanned follow-up

Unplanned follow-up was reported in 187 EmLap patients (36.5%), including representation to the hospital (19.3%), readmission to the hospital (13.3%), and referrals from primary care to specialists (23.2%), with some patients experiencing more than one form of unplanned follow-up. Despite receiving planned surgical outpatient follow-up, 135 patients (26.4%) also required unplanned care after discharge. Among the 99 patients (19.3%) who had unplanned representation to the hospital following discharge, the most common reasons were: wound issues, stoma-related complications, and intra-abdominal sepsis. The 68 patients (13.3%) who required unplanned readmission to the hospital accounted for 68.7% of the patients who represented, indicating that most representations required inpatient admission. Unplanned referrals from primary care to specialists (119, 23.2%) included referrals to: general surgery (49 patients, 9.6%), gastroenterology (31 patients, 6.1%), dieticians (19 patients, 3.7%), and mental health services (15 patients, 2.9%) (Table 4).

**Table 4:** Primary care referrals to specialists after emergency laparotomy

	Site 1 (*N* = 182)	Site 2 (*N* = 103)	Site 3 (*N* = 227)	Total (*N* = 512)
Dietician – no. (%)	4 (2.2)	1 (1.0)	14 (6.2)	19 (3.7)
Gastroenterology – no. (%)	6 (3.3)	7 (6.8)	18 (7.9)	31 (6.1)
Rehabilitation – no. (%)	1 (0.5)	1 (1.0)	7 (3.1)	9 (1.8)
General surgery – no. (%)	1 (0.5)	7 (6.8)	41 (18.1)	49 (9.6)
Mental health – no. (%)	0 (0.0)	2 (1.9)	13 (5.7)	15 (2.9)
Other relevant – no. (%)	4 (2.2)	3 (2.9)	18 (7.9)	22 (4.3)

In cases where patients were referred to multiple services from primary care (England [*n* = 3], Wales [*n* = 19]), each reason was recorded separately.

#### Patient characteristics influencing unplanned follow-up

Multivariable logistic regression analysis demonstrated that a history of mental health (aOR 2.13, 95% CI 1.38–3.29; *p* < 0.001) and prolonged length of hospital stay (aOR 1.90, 95% CI 1.25–2.89; *p* = 0.003) were independently associated with at least one unplanned follow-up event (Table 5).

**Table 5:** Factors associated with unplanned follow-up after emergency laparotomy

Variable	Adjusted odds ratio	95% confidence interval	p-value
Age (per 10-year increase)	0.92	0.82–1.04	0.17
Sex	0.98	0.65–1.46	0.92
Mental Health History	2.13	1.38–3.29	< 0.001
Chronic Pain History	1.68	0.84–3.36	0.14
Cancer Diagnosis	1.11	0.64–1.95	0.71
Stoma Formation	1.22	0.80–1.86	0.37
Prolonged Hospital Stay	1.90	1.25–2.89	0.003

Unplanned follow-up defined as at least one unplanned follow-up event (hospital representation, hospital readmission, or primary care referral to a specialist).

In contrast, increasing age (per 10-year increase) (aOR 0.92, 95% CI 0.82–1.04; *p* = 0.17), sex (aOR 0.98, 95% CI 0.65–1.46; *p* = 0.92), cancer diagnosis (aOR 1.11, 95% CI 0.64–1.95; *p* = 0.71), stoma formation (aOR 1.22, 95% CI 0.80–1.86; *p* = 0.37), and chronic pain (aOR 1.67, 95% CI 0.84–3.36; *p* = 0.14) were not independently associated with unplanned follow-up events.

## Discussion

Exploration of follow-up pathways has highlighted the significant variation in post-discharge care for patients who have undergone emergency surgery. Planned follow-up occurred in only 64.5% of patients, was determined by the responsible surgical team in the absence of standardised guidance, and varied from weeks to months after discharge. Irrespective of planned follow-up, 36.5% of EmLap patients experienced unplanned follow-up, with certain patient factors associated with higher rates of unplanned post-discharge care. This work highlights an urgent need to define, support, and optimise post-surgical care pathways to improve survivorship after EmLap.

Survivorship focuses on addressing the needs of an individual patient as they live with or beyond a life-threatening condition, with the aim of supporting long-term health and quality of life. Although traditionally associated with cancer, the concept of survivorship has extended into other clinical contexts, including intensive care, with the evolution of Post-Intensive Care Syndrome (PICS). This framework addresses new or worsening physical, mental, and neurocognitive disorders following discharge from intensive care and has clear overlaps with addressing our patients’ needs after EmLap [12]. EmLap survivorship remains in the early stages, and this work demonstrates gaps in our understanding of post-operative care pathways and the demands of recovery, which are likely to affect quality of life [6]. EmLap care must be considered beyond discharge to home, with survivorship extending into the years that follow.

Development of a post-operative EmLap pathway will need to be targeted, reflecting the marked heterogeneity of the EmLap population, with older adults living with frailty and multi-morbidity most likely to benefit. This work also identified other factors beyond age that will need to be considered. Patients with mental health conditions or prolonged hospital stays appear to represent groups with greater post-discharge support needs following EmLap, as observed in this cohort. Post-operative pathways will require a multidisciplinary approach, with routinely scheduled follow-up that considers the biopsychosocial needs of patients. Integration of such pathways within existing frameworks, such as NELA and ELLSA, may support their implementation and delivery.

The timing of the planned follow-up may be key to reducing unplanned care. An early post-discharge review, potentially within 1–4 weeks, may help mitigate subsequent unplanned healthcare use, drawing parallels to enhanced recovery programmes in elective surgery, where structure perioperative support and shorter hospital stays have been associated with lower emergency readmission rates [13]. The role of community services may also play a part and is an area to consider analysing in further work.

### Limitations

This study has several limitations. First, three sites were chosen to optimise geographical diversity. Although not representing systems and protocols for all UK sites, the data indicates that the cohort is representative of the NELA and ELLSA populations. Second, hospital representation and readmission were recorded only up to 30 days post-discharge, so the true extent of patient healthcare utilisation is potentially underestimated. Third, this study includes patients who underwent EmLap between 2017 and 2019 and therefore does not capture the potential impact of the COVID-19 pandemic, which has likely exacerbated gaps in follow-up care identified in this study. Fourth, this study did not include patient-reported outcome measures (PROMs), which are increasingly recognised in EmLap survivorship research, thereby limiting the evaluation of recovery from the patient’s perspective. Finally, as an observational study, reported associations should be interpreted as descriptive and hypothesis-generating.

## Conclusions

The findings from this study highlight the complex recovery trajectory following EmLap that continues beyond being discharged home. The observed variation in surgical outpatient follow-up practice and high rates of unplanned follow-up events, indicates a need for future research into defining and intervening on survivorship in the EmLap patient population.

## Supplementary Information

Below is the link to the electronic supplementary material.

Supplementary Material 1.

## Data Availability

The data supporting the findings of this study are available from the corresponding author upon reasonable request.
